# Non-sedation versus sedation with a daily wake-up trial in critically ill patients receiving mechanical ventilation—effects on physical function: study protocol for a randomized controlled trial: a substudy of the NONSEDA trial

**DOI:** 10.1186/s13063-015-0856-1

**Published:** 2015-07-23

**Authors:** Helene Korvenius Nedergaard, Hanne Irene Jensen, Jørgen T. Lauridsen, Gisela Sjøgaard, Palle Toft

**Affiliations:** Department of Anesthesiology and Intensive Care, Lillebaelt Hospital, Skovvangen 2-8, DK-6000 Kolding, Denmark; Department of Business and Economics, Centre of Health Economics research, University of Southern Denmark, Campusvej 55, DK-5230 Odense M, Denmark; Department of Sports Science and Clinical Biomechanics, University of Southern Denmark, Campusvej 55, DK-5230 Odense M, Denmark; Department of Anesthesiology and Intensive Care, Odense University Hospital, Sdr. Boulevard 29, DK-5000 Odense C, Denmark

**Keywords:** Critical illness/rehabilitation, Intensive care, Critical care/methods, Immobilization/physiology, Muscle strength, Muscle weakness/prevention and control, Muscular atrophy/prevention and control, Activities of daily living, Biomechanical phenomena, Quadriceps muscle

## Abstract

**Background:**

Critically ill patients rapidly loose much of their muscle mass and strength. This can be attributed to prolonged admission, prolonged mechanical ventilation and increased mortality, and it can have a negative impact on the degree of independence and quality of life. In the NONSEDA trial we randomize critically ill patients to non-sedation or sedation with a daily wake-up trial during mechanical ventilation in the intensive care unit. It has never been assessed whether non-sedation affects physical function. The aim of this study is to assess the effects of non-sedation versus sedation with a daily wake-up trial on physical function after discharge from intensive care unit.

**Methods/Design:**

Investigator-initiated, randomized, clinical, parallel-group, superiority trial, including 700 patients in total, with a substudy concerning 200 of these patients. Inclusion criteria will be intubated, mechanically ventilated patients with expected duration of mechanical ventilation >24 h. Exclusion criteria will be patients with severe head trauma, coma at admission or status epilepticus, patients treated with therapeutic hypothermia, patients with PaO_2_/FiO_2_<9 where sedation might be necessary to ensure sufficient oxygenation or placing the patient in a prone position. The experimental intervention will be non-sedation supplemented with pain management during mechanical ventilation. The control intervention will be sedation with a daily wake-up trial. The co-primary outcome will be quality of life regarding physical function (SF-36, physical component) and degree of independence in activities of daily living (Barthel Index), and this will be assessed for all 700 patients participating in the NONSEDA trial. The secondary outcomes, which will be assessed for the subpopulation of 200 NONSEDA patients in the trial site, Kolding, will be 6-min walking distance, handgrip strength, muscle size (ultrasonographic measurement of the rectus femoris muscle cross-sectional area) and biomechanical data on lower extremity function (maximal voluntary contraction, rate of force development and endurance).

**Discussion:**

This study is the first to investigate the effect of no sedation during critical illness on physical function. If an effect is found, it will add important information on how to prevent muscle weakness following critical illness.

**Trial registration:**

The study has been approved by the relevant scientific ethics committee and is registered at ClinicalTrials.gov (ID: NCT02034942, 9 January 2014).

## Background

This trial is a substudy in the NONSEDA trial (Clinical Trial identifier: NCT01967680). The aim of the NONSEDA trial is to assess the benefits and harms of non-sedation versus sedation with a daily wake-up trial in critically ill patients in the intensive care unit (ICU). The NONSEDA trial is a multinational trial, where 700 patients will be randomized to non-sedation versus sedation with a daily wake-up trial.

**Fig. 1:**
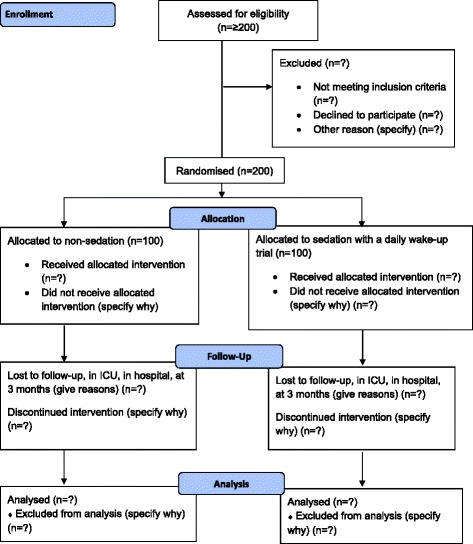
Flowchart. Schematic presentation of the patient flow through the trial

**Fig. 2:**
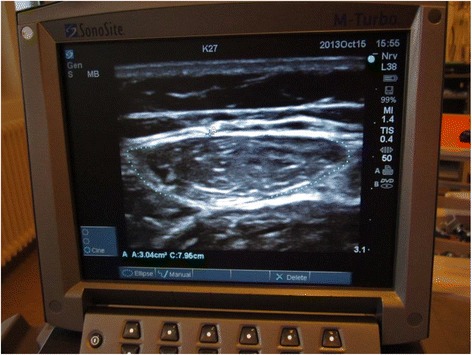
Ultrasonographic measurement of the rectus femoris muscle cross-sectional area. The rectus femoris muscle is part of the quadriceps muscle. Patients will be placed in supine position with their back raised to 45 degrees, with their legs in passive extension. The transducer will be placed over the rectus femoris muscle, perpendicular to the long axis of the right thigh, not depressing the dermal surface. Measurements will be made at 2/3 of the distance from the anterior superior iliac spine to the superior patellar border. This distance will be defined when the patient is placed as noted above, not with the patient standing up, since this changes the distance. For the scan, a linear transducer will be used, flat footprint, 5–8 MHz. The muscle is identified visually and an ultrasonographic picture is taken. Using the ultrasonographic software, the outer edge of the muscle is marked, and the cross-sectional area is calculated using planimetry

**Fig. 3:**
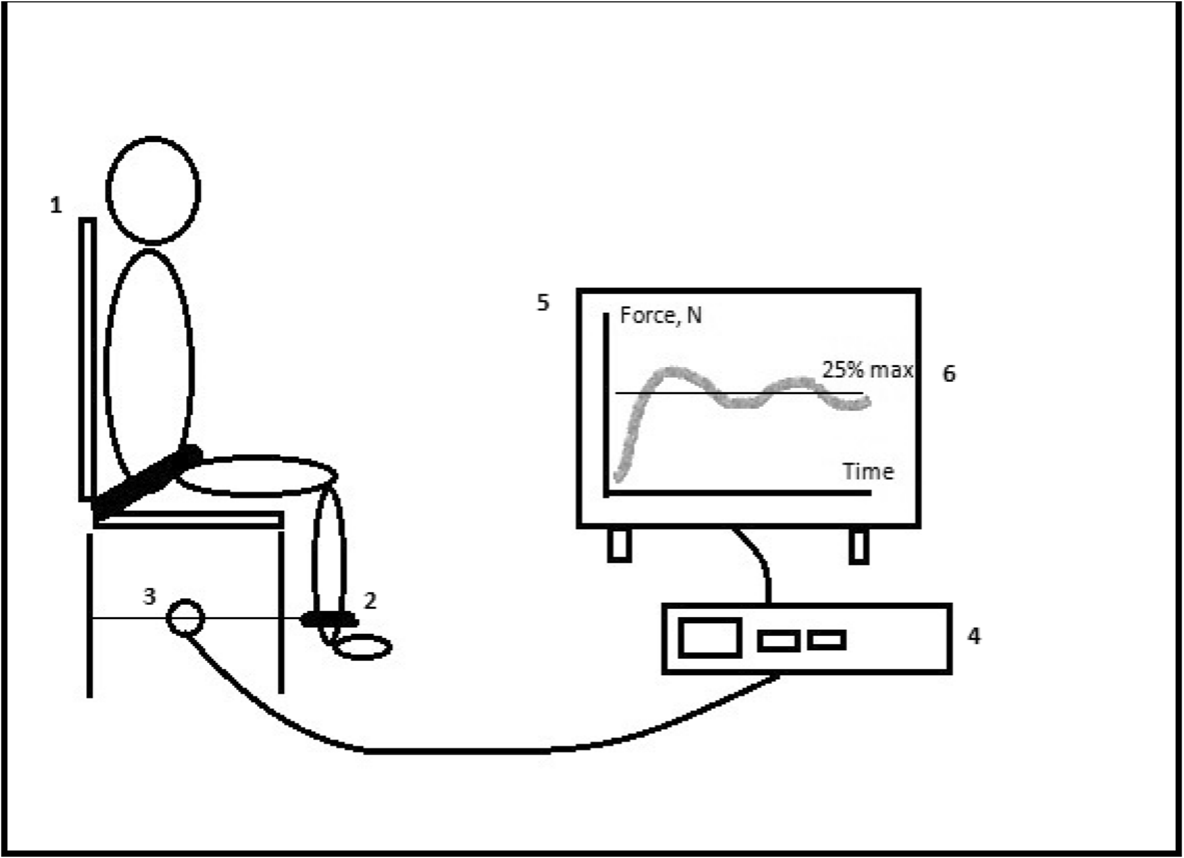
Study setup for biomechanical measurements of the lower limbs. *1*: Custom-made chair, where the participant will be seated, back straight, with 90 degree flexion in the hip, 90 degree flexion in the knee, to ensure that the lower leg is in a vertical position. A safety strap will be fastened at the hip and hands held in the lap. *2*: A strap will be fastened around the ankle, ensuring that the rigid transducer arm (*3*) is horizontal. *3*: Strain-gauge transducer. *4*: Computer with software for data collection. *5*: Screen, where the participant will get visual feedback during endurance testing. A *line* will be marked on the screen (*6*), representing 25 % of the participants’ maximal voluntary contraction (*MVC*). Made with inspiration from J.B. Poulsen and colleagues [47]. The following measurements will be performed: the maximal voluntary contraction (MVC) and rate of force development (RFD). The participant is carefully instructed, using the same wording every time, to “stretch your leg as forcefully and as quickly as possible.” When maximal tension has been reached, it is maintained for 1–2 s and then released. The peak of the force-time curve is the MVC. The steepest slope of the curve is the RFD. *Endurance:* 25 % of the participants’ MVC will be calculated, and a line will be depicted on the screen. The participant will be instructed to exert a force sufficient to reach the line, not more, not less. The participant will maintain this force for as long as possible, though maximally 3 min. The participant will have constant visual feedback and standardized verbal encouragement, if needed

**Fig. 4:**
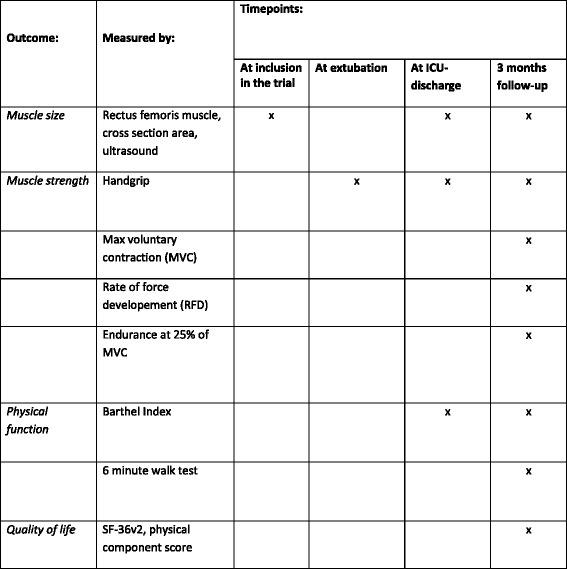
Outcomes and time points. The primary, secondary and exploratory outcomes and relevant time points

Two hundred of the 700 patients in the NONSEDA trial will be included, treated and followed up in the intensive care unit in Kolding. This protocol describes a substudy concerning physical function (quality of life regarding physical function, walking ability, ability to perform everyday tasks, biomechanical data and muscle size) and will be based on these 200 patients.

### Patient population

In Denmark approximately 30,000 patients (2-3 % of all hospital patients) are admitted to ICUs every year. In 2011 mortality during the ICU stay was 12.7 % and 30-day mortality 21.2 % [1]. An intensive care admission can have substantial consequences for patients, and studies show that ICU survivors have a decreased quality of life and increased mortality for years after discharge [2].

### Current care and treatment

Muscle wasting and ICU-acquired weakness is frequently encountered in the ICU, affecting up to 25 % of patients requiring mechanical ventilation for more than a week and more than half of the patients suffering from severe sepsis [3, 4]. Muscle mass reduces rapidly, with severely septic patients losing 15-20 % within the first week [3]. It is a condition with high costs for both the patient and the healthcare system, since it is associated with increased length of stay in the ICU and hospital, prolonged mechanical ventilation and increased in-hospital mortality, and it affected the quality of life for months or even years after discharge [2, 3, 5–7].

Since the dawn of ventilator therapy, it has been standard care to sedate the patients continuously. The first ventilators were rather primitive and very uncomfortable for the patients, making the sedation practice necessary. As ventilators have become increasingly advanced, and now allow for high patient-ventilator interaction and relative comfort, the focus has been on a lighter level of sedation. Numerous trials have documented beneficial effects of less sedation, namely shorter duration of mechanical ventilation, lower morbidity and shorter length of stay in the ICU and hospital [8–14]. Ali and colleagues have demonstrated an association between the time patients spend in coma and muscle wasting and ICU-acquired weakness [7, 15]. Schweikert and colleagues carried out a randomized, clinical trial of sedation interruptions combined with early physiotherapy for mechanically ventilated, critically ill patients [16]. Their intervention group had more ventilator-free days and a better functional outcome at hospital discharge. Several trials have demonstrated that it is both feasible and safe to mobilize mechanically ventilated ICU patients [17].

It is appealing to speculate that less sedation will lead to more alert and thereby more physically active patients, who can be mobilized to a larger extent [18]. However, it has not been established whether physical function following ICU discharge is affected by non-sedation versus the standard care of sedation with a daily wake-up trail.

In the NONSEDA trial protocol we did at systematic literature search, which established that, for the present (October 2014), only one randomized clinical trial on the effect of non-sedation versus sedation with a daily wake-up trial has been published, namely the aforementioned trial from Odense University Hospital [9]. This trial did not investigate physical function.

### Trial conduct

The protocol has been approved by The Regional Scientific Ethical Committees for Southern Denmark (protocol ID: S-20130025). We will obtain informed consent from the patients who are sufficiently awake; otherwise, the informed consent will be obtained from the closest relative and the patient’s general practitioner, alternatively the Medical Health Office.

The trial is registered at ClinicalTrials.gov, ID: NCT02034942 (9 January 2014).

The trial has been approved by the Data Protection Agency (#2008-58-0035, approval for the Region of Southern Denmark).

### Trial objective

The objective will be to assess the benefits and harms of non-sedation versus sedation with a daily wake-up trial in critically ill patients concerning physical function following ICU-discharge.

### Trial hypothesis

The primary hypothesis is that non-sedation compared with sedation and a daily wake-up trial will lead to less affected physical function following ICU discharge.

## Design

The trial is a substudy in the NONSEDA trial (ClinicalTrial ID: NCT01967680). The NONSEDA trial is an investigator-initiated, randomized, clinical, parallel-group, multinational, superiority trial designed to include 700 patients from at least six intensive care units in Denmark, Norway and Sweden. Two hundred of the 700 patients will be included, treated and followed up in the ICU in Kolding. This substudy will be based on these 200 patients (see section “Outcomes”) Fig 1.

### Randomization

Patients will be randomized to one of the two groups within 24 h after intubation. If the patient arrives intubated from another ICU, the patient will be randomized within the first 24 h after arrival. The randomization will be carried out centrally by the Copenhagen Trial Unit according to a computer-generated allocation sequence with a variable block size, kept concealed from the investigators at the clinical sites. The allocation sequence will be stratified by center, age (up to 65 years or older) and shock at admission (systolic blood pressure <70 mmHg or above). The 200 patients for this substudy will be all the patients included in the Kolding trail site. Since we will stratify for center, we will obtain an equal distribution of the patients. The randomization system will be Internet based with 24 h access a day, 7 days a week. The participant allocation will be carried out by an investigator who logs in to the CTU’s Online Randomization System using a personal ID and PIN code. Then the investigator will type in all relevant information about the participant, and the participant will be subsequently allocated to either the ‘non-sedation group’ or the ‘sedation group.’

### Blinding

Due to the nature of the trial interventions, it will not be possible to blind the investigators at clinical trial sites, the participants or the participants’ relatives. All other parties in the trial will be blinded. The statistical analyses will be conducted blinded with the two intervention groups coded as, e.g., A and B.

Concerning the two co-primary outcomes in this substudy (the SF-36 and Barthel Index), all information about the questionnaire will be standardized and similar for all participants, and the research assistant handling the data will be unaware of the patient’s treatment allocation. If, in rare circumstances, it is necessary to perform the questionnaires as a telephone interview, this will be done by an investigator from another trial site than where the patient was treated; the assessor will thus be blinded.

### Inclusion criteria

- Endotracheally intubated
- Expected time on ventilator >24 h
- Age ≥18 years
- Informed consent

### Exclusion criteria

- Severe head trauma where therapeutic coma is indicated
- Therapeutic hypothermia where therapeutic coma is indicated
- Status epilepticus where therapeutic coma is indicated
- The patient has participated in the study before
- The patient was transferred from another ICU with length of stay >48 h
- The patient is comatose at admission
- PaO_2_/FiO_2_ ≤ 9, where sedation might be necessary for oxygenation

### Trial site and personnel

The Intensive Care Unit, Lillebaelt Hospital, Kolding, is a mixed medical and surgical ICU with 11 ICU beds and 3 intermediate care beds. The unit treated 850 patients in 2012 and 894 patients in 2013. The trial personnel will be doctors, nurses and physiotherapists working in the Kolding ICU. The personnel are already used to working with non-sedation and handling awake, mechanically ventilated patients as well as sedated patients with daily wake-up trials. The trial group will monitor the clinical work and, if needed, provide supplementary training in non-sedation and daily wake-up trials, both in theory and by supervised practice.

### Interventions

As described in the NONSEDA trial protocol, the intervention consists of non-sedation versus sedation with a daily wake-up trial. In this substudy we will investigate how non-sedation versus sedation with a daily wake-up trial affects physical function after ICU discharge. For details about the interventions, see the NONSEDA trial protocol, Clinical Trial identifier NCT01967680 (www.clinicaltrials.gov).

### Co-interventions

As stated in the NONSEDA trial protocol, both groups will receive analgesic treatment as usual, with opiates and paracetamol, in order to keep patients comfortable. In case the patient arrives at the department with an epidural catheter, the analgesia will continue via the epidural catheter as usual. The Numeric Rating Scale (NRS, 0–10) will be used to monitor the need for supplemental analgesics, and morphine will be given if the NRS is ≥ 3 at rest and ≥5 during activity.

Mobilization and physical activation of the patients are not done according to a protocol, but are carried out in accordance with the local standards of the Kolding ICU. We will register the amount of passive and active mobilization for each patient on a daily basis, since one can speculate that the more awake a patient is, the more they will be mobilized.

We have defined three co-interventions, which are suspected of being associated with the development of ICU-acquired weakness, although the results of trials have been contradictory [5, 19–21]. They will be registered and presented for each intervention group:

- Use of neuromuscular blocking agents
- Use of corticosteroids
- Blood glucose profiles

The co-interventions defined in the NONSEDA trial protocol are use of vasoactive agents and antibiotics as well as total amount of fluids given. These co-interventions are not thought to have a direct effect on muscular function, which is why we have defined separate co-interventions for this substudy.

### Outcomes

Outcomes on physical function will be investigated for the two different patient populations separately, namely the total population of all 700 patients in the NONSEDA trial and the 200 patients in the substudy from the Kolding trial site. The primary outcome of this substudy will be based on the total population of 700 patients and will be a co-primary outcome of:

- patient-reported quality of life, as measured by SF-36v2, physical component score [22, 23] and degree of independence in activities of daily living, as measured by the Barthel Index [24–26].

The secondary outcomes of this substudy will be based on the subpopulation of 200 patients participating in the substudy in Kolding and will be:

- Walking distance in the 6-min walk test at 3-month follow-up [27–32]
- Handgrip strength at 3-month follow-up [7, 33–35]
- Barthel Index at discharge from the ICU
- Muscle size, measured as cross-sectional area of the rectus femoris muscle (see Fig. 2) at 3-month follow-up [36]
- Muscle strength, measured using biomechanical measurements−maximal voluntary contraction (MVC), rate of force development (RFD) and endurance at 25 % of the MVC (see Fig. 3) at 3-month follow-up [37–40].

Exploratory outcomes will be:

- Handgrip strength at extubation
- Handgrip strength at ICU discharge
- Muscle size, measured as cross-sectional area of the rectus femoris muscle, at extubation
- Muscle size, measured as cross-sectional area of the rectus femoris muscle, at ICU discharge Figs. 2, 3 and 4.

### Safety

There is no known risk associated with participation in the substudy. As a part of the NONSEDA protocol we register accidental extubation requiring re-intubation within an hour and accidental removal of the central venous line requiring reinsertion within 4 h as serious adverse events.

### Inclusion of patients

Patients can be admitted to the ICU either from the same hospital (emergency department or another ward) or transferred from an ICU in another hospital. If they are admitted from within the same hospital, they are either not intubated or have been intubated within a very short time, for example during pre-hospital care. Patients will be included in the study within 24 h from intubation.

Patients transferred from an ICU in another hospital are very often intubated. If they are transferred from another ICU, they can be included in the trial if the stay in the other ICU was shorter than 48 h. In the time leading up to inclusion and randomization, it will vary whether patients are sedated or not, depending on the particular clinician on duty and traditions at the particular hospital.

### Obtaining informed consent

When patients are contacted the first time concerning participation in the study, they will be at the ICU. Verbal and written information will be given by one of the participating physicians or study nurse. Patients are informed about the rights to assistance and the possibility of reflection time. Patients will be considered competent if they are awake and not delirious (negative CAM-ICU). The competent patients will give consent after a period of reflection time of up to several hours. If patients are not awake and not competent because of their illness, surrogate consent will be obtained from a close relative and the patient’s private practitioner, alternatively the Medical Health Office. The consent of a relative relies on the patient’s presumed attitude to participation in clinical trials. The connection between the relative and the patient will appear in the surrogate consent form. Like the patient, the relative is also given time of up to several hours to make the decision.

If a patient or the relatives no longer wish to participate in the trial for any reason, they will be asked for permission to use the already obtained data, to obtain data from electronic patient files for the rest of the trial period and to invite the patient to the 90-day follow-up.

### Data collection

The table in Fig. 4 shows the types of data and the time the data will be collected. If not otherwise stated, data originate from medical records included in the Critical Information System (CIS) or other electronic patient files.

The SF 36v2 questionnaire and the Barthel Index will be sent to the participant, who will fill it out at home and bring it to the follow-up. If a participant has not filled out the questionnaire before the follow-up, the participant will be asked to complete the questionnaire at follow-up. They will be seated in privacy in a calm place, with sufficient time to fill out both questionnaires (at least 30 min).

In an effort to minimize missing data as much as possible, both the SF-36v2 and Barthel Index can be performed as a telephone interview. Telephone interviews are a last resort, and every effort will be made to ensure that as many patients as at all possible fill out the questionnaires themselves. SF-36 can only be carried out as an interview with the participant in person, since it concerns the person’s self-perceived quality of life. The Barthel Index is preferably performed as an interview with the participant in person, but if no other option exists, it can be carried out with a proxy instead. Therefore, if a participant for some reason is unable to participate in the follow-up and unable to fill out the questionnaire, a telephone interview will be performed. It is estimated that a telephone interview will take approximately 20 min.

Measurements of muscle size and biomechanical data will be obtained by investigator HKJ and a trial nurse. If the participant is not able to come to the hospital for follow-up, we will ask if we can come to their home to do the follow-up. The 6-min walk test can be performed on an outdoor, marked 30-m trail at the participant’s home if they are able to walk. Ultrasonographic measurements of muscle size can likewise be performed at the participant’s home. Biomechanical data can only be collected in the hospital because the equipment is very difficult to move.

Before contacting any patient after discharge, we will check with national central person registrations to assure that the patient is not deceased. In summary, the process for establishing the follow-up will be as follows: Approximately 14 days prior to the 3-month follow-up we will send a letter to the patient, containing the invitation to the follow-up and the questionnaires to be filled out (SF-36 and Barthel Index). If the patient does not respond within 10 days, we will phone the patient. We will first repeat the invitation to participate in the follow-up and clarify any misunderstandings concerning payment for transportation or the like. If the patient declines to come to the hospital, we will offer to come to the patient’s home and do the follow-up there (6-min walk test and muscle size). If the patient declines this, we will ask if we can perform the SF-36 and Barthel Index over the telephone.

### Data management

An electronic Case Record Form (eCRF) for the NONSEDA trial in Open Clinica is developed in cooperation between the coordinating investigator and a data manager at Copenhagen Trial Unit. Access to the eCRF will be possible around the clock every day where data continuously can be entered for all the randomized patients.

The coordinating investigator will have access to monitor the data input. If the entry is partially or completely missing or seems flawed on one or more randomized patients, the coordinator will have the opportunity to contact the primary investigator in order to correct or complement data inputs to optimize the quality of the data.

### Power estimation

The sample size estimation for the NONSEDA study will be 700 patients, with 350 in each group [please see the NONSEDA trial protocol, Clinical Trial identifier NCT01967680 (www.clinicaltrials.gov) for details]. These sample size calculations were made using the power and sample size program PS [41].

Estimation of power for the co-primary outcome: We will have a sample of 350 experimental participants and 350 control participants. Based on similar study populations, we estimate 40 % mortality [42, 43]. The study population for co-primary outcome analyses will therefore be a total of 420 patients, with 210 in each group. Although every effort will be made to obtain as high a response rate as at all possible, we have to expect some degree of non-responders, which we estimate will be 10 %. That leaves 378 patients, 189 in each group.

- SF-36, physical component score: We expect that the response within each subject group is normally distributed with a standard deviation of 20 and a minimal clinically relevant difference of 5 [44]. Since this is the first study to investigate the effect of non-sedation on physical function, it is unknown how large an effect can be expected. If the true difference in the experimental and control means is 5, we will be able to reject the null hypothesis that the population means of the experimental and control groups are equal with a probability (power) of 0.57. If the true difference is 7, we will be able to reject the null hypothesis with a probability (power) of 0.87. The type I error probability associated with this test of this null hypothesis is 0.025.
- Barthel Index: We expect that the response within each subject group is normally distributed with a standard deviation of 5 and a minimal clinically relevant difference of 2 [45]. If the true difference in the experimental and control means is 2, we will be able to reject the null hypothesis that the population means of the experimental and control groups are equal with a probability (power) of 0.95. The type I error probability associated with this test of this null hypothesis is 0.025.

Estimation of the power for the main secondary outcome: We will have a sample of 100 participants and 100 control participants. We estimate 40 % mortality. The study population for the main secondary outcome will therefore be a total of 120 patients with 60 in each group. Since this group of patients is fragile and often elderly, and since participation in the follow-p requires an hour of active participation and (most often) a trip to the hospital, we estimate that 25 % will not participate in the follow-up, leaving 90 patients, 45 in each group.

- Six-minute walk test: We expect that the response within each subject group is normally distributed with a standard deviation of 90 [31] and a minimally clinically relevant difference of 55 m [30]. If the true difference in the experimental and control means is 55, we will be able to reject the null hypothesis that the population means of the experimental and control groups are equal with a probability (power) of 0.82. The type I error probability associated with this test of this null hypothesis is 0.05.

### Statistical analysis plan

All continuous normally distributed outcome data will be described by the mean, mean difference, standard deviation (SD) and range. Non-normally distributed outcome data will be described by the median and interquartile range. Student’s *t*-test and multiple regression will be used to analyze the differences between the two groups. For non-normally distributed data non-parametric analyses such as the Mann-Whitney U-test will be used. Multiple regression will be used to handle repeated measurements. We will perform intention-to-treat analyses.

All patients are followed up for at least 3 months after discharge via the electronic eCRF, Social Security Register and the National Patient Register. Missing data will be handled in accordance with multiple imputation procedures if missing data are greater than 5 % and Little’s test is statistically significant [46]. The imputation result will be considered the primary overall result but per protocol analyses will also be presented.

All raw *p*-values and confidence intervals of all outcome comparisons between the two groups will be presented. A *p*-value < 0.05 will be considered statistically significant in all analyses, except for co-primary outcome, where a *p*-value < 0.025 will be considered statistically significant. Statistical analysis of the data will be done using STATA 13.

## Discussion

The purpose of this randomized controlled trial is to investigate whether the degree of sedation during ventilator treatment affects physical function after discharge from the ICU, with self-perceived quality of life concerning the physical function and degree of independence in every activity as primary outcome.

The literature on how non-sedation affects physical function is very limited, since the tradition as well as current golden standard is a higher degree of sedation, although, for the time being, there is a growing interest in limiting sedation and applying early mobilization and physiotherapy in the ICU. Having non-sedated patients on the ventilator seems to increase the amount of physical activity not only during formal physiotherapy sessions, but also for the rest of the days, where the patient is able to move around in a bed or chair to a greater extent. Whether this plays a role in the physical status after discharge is unknown.

Since this trial is a part of a larger trial, where sedation with a daily wake-up call versus non-sedation is the intervention, there is no separate intervention concerning physical function. Since it is obvious to speculate that the non-sedated patients will be mobilized to a larger extent, we will quantify the degree of physical activity every day, both active (e.g., standing on the floor, bed-biking) and passive (e.g., passive movements of the limbs by the physiotherapist). However, all the small movements a non-sedated patient might make during the day cannot be quantified. Taking other possible confounders into concern, e.g., blood sugar, use of neuromuscular blocking agents or steroids, this might be part of the explanation if non-sedated patients prove to have a better physical status. These factors will also be quantified.

ICU survivors are a group of mainly elderly patients with a high degree of comorbidity. This complicates the follow-up investigation, since some patients will be too weak to participate. As secondary and exploratory outcomes, we try to obtain data on physical function already at the time of extubation and at ICU discharge. This might prove to be futile, since patients at these time points are far from recovered. With two pilot patients we attempted to obtain biomechanical data on lower limb function at ICU discharge, but we had to realize that the patients at this time point are not in a condition where this is possible. In an attempt to obtain early data that are not depending on patient cooperation, we measure the muscle size of the rectus femoris muscle and thereby quantify the amount of muscle wasting in the two groups. What could complicate these measurements is the degree of edema, which ICU patients (especially septic patients) are prone to. It does seem reasonable to assume that the degree of edema will be equal between the two groups at the time of inclusion and at the time of extubation and discharge. We are also aware that the mere size of a muscle does not in itself translate to function, and muscle size must be regarded as a surrogate parameter.

Poulsen and colleagues in 2013 published results on biomechanical function in ICU survivors (males, aged 50–75 years, 12 months after ICU discharge) compared to healthy, age- and sex-matched controls [47]. Their study is the first to provide data on biomechanical data in ICU survivors and, to the best of our knowledge, still the only one. Our setup is in many respects similar to theirs, hopefully providing the basis for comparisons. Among others, Poulsen et al. found that especially the rate of force development was negatively affected in ICU survivors. The rate of force development is known to be an important factor in activities of daily living since it is crucial for postural stability. The rate of force development can be improved with specific training programs. Should our study support this finding or reveal other characteristics of muscles recovering after critical illness, it could provide further basis for targeted physiotherapy interventions.

>The validity of this trial is affected by the fact that it is not blinded, but blinding is not possible with this intervention. We find that the very realistic clinical context the trial is set in—broad inclusion criteria, mixed ICU and numerous caregivers—increases its generalizability and external validity.

We find that the use of sedatives for critically ill patients is still affected by tradition and habits, and it varies from department to department. With the NONSEDA trial we hope to shed light on the possible benefits or harms of being non-sedated. Non-sedation is a complex intervention and has numerous possible consequences, and all of these deserve to be clarified in order to obtain a nuanced picture of whether non-sedation is the way forward. One of these possible consequences is the effect on physical function.

## Trial status

The trial is now actively recruiting patients. We have included 96 of the 200 patients (July 2015).
